# Intercropping of tobacco and maize at seedling stage promotes crop growth through manipulating rhizosphere microenvironment

**DOI:** 10.3389/fpls.2024.1470229

**Published:** 2024-10-09

**Authors:** Junmei Ma, Di Liu, Peiyan Zhao, Min Dou, Xiuhua Yang, Shulei Liu, Fuzhao Nian, Wenjie Tong, Junying Li, ZhaoLi Xu, Liuchen Zhang, Hong Zhang, Yongzhong Li, Xiaopeng Deng, Yating Liu

**Affiliations:** ^1^ College of Tobacco Science, Yunnan Agricultural University, Kunming, Yunnan, China; ^2^ College of Plant Protection, Yunnan Agricultural University, Kunming, Yunnan, China; ^3^ College of Resource and Environment, Yunnan Agricultural University, Kunming, Yunnan, China; ^4^ Yunnan Academy of Tobacco Agricultural Sciences, Yunnan Tobacco International Co., Ltd, Kunming, Yunnan, China

**Keywords:** intercropping, crop growth, soil nutrients, microorganisms, metabolites

## Abstract

**Introduction:**

Changes in the rhizosphere microbiome and metabolites resulting from crop intercropping can significantly enhance crop growth. While there has been an increasing number of studies on various crop combinations, research on the intercropping of tobacco and maize at seedling stage remains limited.

**Methods:**

This study is the first to explore rhizosphere effects of intercropping between tobacco and maize seedling stages, we analyzed the nitrogen, phosphorus and potassium nutrients in the soil, and revealed the important effects on soil microbial community composition and metabolite profiles, thereby regulating crop growth and improving soil balance.

**Results and discussion:**

Compared with mono-cropping, intercropping increased the biomass of the two crops and promoted the nutrient absorption of nitrogen, phosphorus and potassium. Under intercropping conditions, the activities of sucrase, catalase and nitrate reductase in tobacco rhizosphere soil and the content of available potassium, the activities of nitrate reductase and acid phosphatase in maize rhizosphere soil were significantly increasing. Rhizosphere soil bacterial and fungal communities such as Sphingomonas, Massilia, Humicola and Penicillium respond differently to crop planting patterns, and soil dominant microbial communities are regulated by environmental factors such as pH, Organic Matter, Available Potassium, Nitrate Reductase, and Urease Enzyme. Network analysis showed that soil microbial communities were more complex after intercropping, and the reciprocal relationship between bacteria and fungi was enhanced. The difference of metabolites in soil between intercropping and monocropping system was mainly concentrated in galactose metabolism, starch and sucrose metabolism pathway, and the content of carbohydrate metabolites was significantly higher than that of monocropping soil. Key metabolites such as D-Sucrose, D-Fructose-6-Phosphate, D-Glucose-1-Phosphatel significantly influence the composition of dominant microbial communities such as Sphingomonas and Penicillium. This study explained the effects of intercropping between flue-cured tobacco and maize on the content of soil metabolites and soil microbial composition in rhizosphere soil, and deepened the understanding that intercropping system can improve the growth of flue-cured crops seedlings through rhizosphere effects.

## 1 Introduction

Intercropping is an intensive agricultural practice that has garnered increased attention for its potential benefits (Rakotomalala et al., 2023). Recent research has focused on the interactions of crop roots and the competition for soil moisture, nutrients, and space in intercropping systems. The plant rhizosphere plays a crucial role in the uptake of nutrients, water, and chemicals by crops, acting as a dynamic interface for material exchanges within the soil-plant ecosystem (Xu et al., 2023). Crops grown in intercropping systems typically exhibit larger belowground biomass and more diverse rhizosphere soil microenvironments. This diversity enhances the secretion of soil metabolites, microbial activity, and nutrient cycling (Tang et al., 2015).

Soil metabolites primarily originate from plant root secretions, rhizosphere microbial processes, and soil organic matter decomposition (Zhang et al., 2020). These compounds serve as carbon sources and signaling molecules for rhizosphere microbiome, influencing the microbial community structure and playing a vital regulatory role in soil physicochemical properties, crop growth, and development (Massalha et al., 2017; Gao et al., 2023). Soil microbiome are essential for nutrient transformation, cycling, system stability, resilience to disturbances, and sustainable resource utilization (Liu et al., 2014; Bitem et al., 2020). Positive interactions among species in intercropping systems are achieved by creating beneficial soil microbial communities influenced by root-secreted substances (Li and Wu, 2018).

In intercropping systems, rhizosphere microbiome facilitate soil organic matter decomposition and nutrient transformation, enhance nutrient uptake by plants, and affect plant growth processes (Zaeem et al., 2019). For instance, in a peanut and cassava intercropping system, cyanide released by cassava roots can induce ethylene production in peanut roots. This modifies the composition of rhizosphere microbial communities and substantially increases the α diversity of peanut rhizosphere bacteria and the relative abundance of rhizosphere actinomycetes compared to monoculture (Tang et al., 2020). Plant diversity also significantly affects soil microbial communities (Cappelli et al., 2022). Intercropping enhances the abundance of rhizosphere soil bacteria and fungi compared to monoculture, improves soil microbial activity, and alters the microbial community structure (Dong et al., 2019; Fu et al., 2021).

Maize (Zea mays L.) is a vital grain crop and can also be used as silage. Tobacco (Nicotiana tabacum L.) is an important model crop for biological research and is the initial raw material for industrial and medicinal products. It is often rotated and intercropped with maize in Southwest China. Tobacco has specific growth requirements and a complex root environment control system. Few studies have comprehensively analyzed the mechanisms of rhizosphere-metabolite-mediated changes in microbial diversity and functional capacity in tobacco and maize intercropping systems. This study aims to deepen the understanding of the intercropping-driven advantages in tobacco and maize growth through a pot experiment assessing the effects of tobacco-maize intercropping and analyzing root interactions between the two crops. The specific objectives are to: (1) clarify the variation in rhizosphere metabolite profiles between different crop planting systems and identify key metabolite components, (2) determine the relationships between rhizosphere metabolites and microbial diversity and functional microbial populations, and (3) understand how different planting patterns regulate crop growth and nutrient utilization via rhizosphere processes and management.

The findings of this research will enhance our understanding of the interrelationships among plants, soil microorganisms, and rhizosphere soil metabolites in intercropping systems, provides new references for crop selection and arrangement of crop planting patterns in intensive planting systems, offers a scientific basis for sustainable agricultural development, to achieve the purpose of enhancing crop productivity.

## 2 Materials and methods

### 2.1 Experimental site and basic agrochemical characteristics of soil

The study was conducted from March to May 2023 in the greenhouse of the experimental base in Yunnan Agricultural University in Kunming, China. The average daily temperature in the greenhouse was 25 °C, with an average daily light intensity of 1650 μmol·m^-2^·s^-1^, the average relative environmental humidity was approximately 74% and the vapor pressure deficit was around 0.814 kPa. The experimental soil was red soil, with the following agrochemical characteristics: alkaline nitrogen content of 86.4 mg/kg, available phosphorus content of 22.5 mg/kg, quick-acting potassium content of 328.57 mg/kg, organic matter content of 18.0 g/kg, and pH value of 6.8.

### 2.2 Experimental design

The research objects are the tobacco variety “K326” and the corn variety “Yunrui 319.” Three different treatments were set up: K326 monoculture (KT), Yunrui 319 monoculture (YC), and K326 intercropped with Yunrui 319 (KYI). A pot experiment with plastic flower pots (50 cm × 20 cm × 18 cm) was conducted. The experimental soil was sieved through a 5 mm sieve and thoroughly mixed with vermiculite at a volume ratio of 3:1. Each pot was filled with 9 kg of the mixture, with 15 replicates in each treatment. For the intercropping treatment of tobacco and maize, one tobacco plant and one maize plant were planted in each pot with a spacing of 30 cm. For the monoculture treatment of tobacco or maize, two tobacco or maize plants were planted in each pot with a spacing of 30 cm. Tobacco seedlings were transplanted after a bed period of 45 days. The maize seeds were aseptically germinated 7 days before transplanting the tobacco plants and were sown at a depth of 3-5 cm. During the experiment, each pot was fertilized with 13.67 g of compound fertilizer (N-P_2_O_5-_K_2_O=10-10-25, purchased from Yunnan Yunye Fertilizer Co., Ltd.) on days 1, 15, and 30 after transplanting. The fertilizer (13.67 g each time), was dissolved in distilled water and evenly applied to the pots (calculated based on 4.5 kg of pure nitrogen per 1100 plants/667 m^2^ in the field). Additionally, consistent practices of watering, fertilization, weeding, and pest and disease control were maintained. Samples were collected for analysis 40 days after planting.

### 2.3 Sample collection and parameter determination

#### 2.3.1 Soil sample collection and biomass determination

The plants were uprooted with soil, wash cleaned, and weighed to determine the fresh weight of the aboveground and belowground parts. Subsequently, the plant samples were withered at 105°C for 30 minutes and oven-dried at 80°C to calculate the dry weight of both parts.

#### 2.3.2 Soil sample collection

Carefully scraping off the soil around the roots (0-5mm) and passing it through a 2mm screen, rhizosphere soil samples of intercropped tobacco (K326) and maize (Yunrui 319) and monoculture tobacco and maize were collected. The fresh rhizosphere soil samples were frozen rapidly in liquid nitrogen for 30 minutes and stored at -80°C for subsequent high-throughput microbial sequencing and metabolite analysis. Another portion of the rhizosphere soil was air-dried and sieved through a 40-mesh sieve to analyze soil agrochemical properties and enzyme activities.

#### 2.3.3 Rhizosphere soil nutrient and enzyme activity measurement

The soil pH was determined using water extraction, and the organic matter content was analyzed by potassium dichromate titration. The alkali nitrogen content was assessed by alkali diffusion, and the available phosphorus content was measured using NaHCO_3_ extraction-molybdenum antimony colorimetry. The available potassium content was determined by ammonium acetate extraction-flame photometry. We followed the procedures outlined in the “Soil Agrochemical Analysis” (Bao, 2005). Soil sucrase(G0302W), catalase(G0303W), urease(G0301W), nitrate reductase(G0309W), and acid phosphatase(G0304W) activities were measured with reagent kits provided by Suzhou Grace Biotechnology Co., Ltd (Khan et al., 2022).

#### 2.3.4 Plant uptake and cumulative amounts of nitrogen, phosphorus, and potassium

After drying, grinding, and sieving the plant parts, the nitrogen, phosphorus, and potassium contents were measured separately for aboveground and belowground parts following the NY/T 2017-2011 standard “Plant Nitrogen, Phosphorus, and Potassium Determination Methods”.

#### 2.3.5 Analysis of rhizosphere soil microbial diversity

The rhizosphere soil samples were sent to Shanghai Paiseno Biotechnology Co., Ltd., Nanjing, Jiangsu for DNA extraction, polymerase chain reaction (PCR) amplification, library construction, and high-throughput sequencing. The universal primers F: 5’- ACTCCTACGGGAGGCAGCA-3’ and R: 5’- GGACTACHVGGGTWTCTAAT-3’ were employed for PCR amplification of the bacterial 16S rRNA gene 16S_V3V4a region, and the universal primers F: 5’- GGAAGTAAAAGTCGTAACAAGG-3’ and R: 5’- GCTGCGTTCTTCATCGATGC-3’ were used for PCR amplification of the fungal ITS gene ITS_V1 region. Raw sequencing data were deposited in the NCBI Sequence Read Archive (SRA, https://submit.ncbi.nlm.nih.gov/subs/sra/) with accession No. PRJNA1105019 and No. PRJNA1104889.

#### 2.3.6 Metabolite profiling of rhizosphere soil

Soil metabolites were extracted using a comprehensive targeted metabolomics analysis conducted by Wuhan Metware Biotechnology Co., Ltd. 50 mg soil sample was thawed on ice and then homogenized with 500 μl of ice-cold methanol/water (70%, v/v). The samples were incubated on ice for 15 minutes and then centrifuged at 12,000 rpm at 4°C for 10 minutes. To the precipitate, 500 μl of ethyl acetate/methanol (1:3, v/v) was added, and the mixture was shaken for 5 minutes, followed by another incubation on ice for 15 minutes. The mixture was then centrifuged again at 12,000 rpm at 4°C for 10 minutes, and 400 μl of the supernatant was collected. The two supernatants of 800 μl were then combined and concentrated, subsequently dried, and reconstituted with 100 μl of 70% methanol in water. The solution was ultrasonicated for 3 minutes. Finally, it was centrifuged at 12,000 rpm for 3 minutes at 4°C, and 60 μl of the supernatant was extracted for liquid chromatography-tandem mass spectrometry (LC-MS/MS) analysis. The detection of soil metabolites was performed using an ultra-high-performance liquid chromatography system (Agilent 7890, Santa Clara, CA, USA) coupled with a mass spectrometer (QTOF/MS-6545, LECO, St. Joseph, MI, USA). Data processing was performed using ProteoWizard software and the XCMS package. Subsequently, R software (version 1.6.2) was used for further data analysis with the ropls package. Significant differential metabolites were identified based on Orthogonal Partial Least Squares Discriminant Analysis (OPLS-DA) model variable importance.

### 2.4 Data processing and analysis

The soil physical and chemical properties, and enzyme activities were managed by Excel 2016, independent sample T-tests and correlation analysis were conducted using SPSS 26.0, and the graphs were created in Origin 2020.

The optimized sequences were clustered at a 97% similarity threshold using the Paiseno Genes Cloud platform (https://www.genescloud.cn/login) to analyze the diversity of rhizosphere soil microbiota. Microbial species were identified, the sequencing depth and coverage was assessed, and the differences of bacterial and fungal communities in crop roots between different treatments were compared. Core microbiota was evaluated, and the correlation analysis of microbial communities and environmental factor were investigated by correlation Co-occurrence network data analysis for bacteria and fungi was conducted and visualized using Gephi 0.9.3.

Orthogonal partial least squares discriminant analysis (OPLS-DA) was used to analyze the rhizosphere soil metabolites using the Metware platform (https://cloud.metware.cn). Variance analysis and OPLS-DA were employed to identify key differential metabolites (*P* < 0.05 and VIP > 1). Environmental factor correlation analysis was used to assess the factors influencing changes in metabolites, followed by correlation analysis of the dominant microbiota.

## 3 Results and analysis

### 3.1 Effects of intercropping on the growth of tobacco and maize

**Figure 1:**
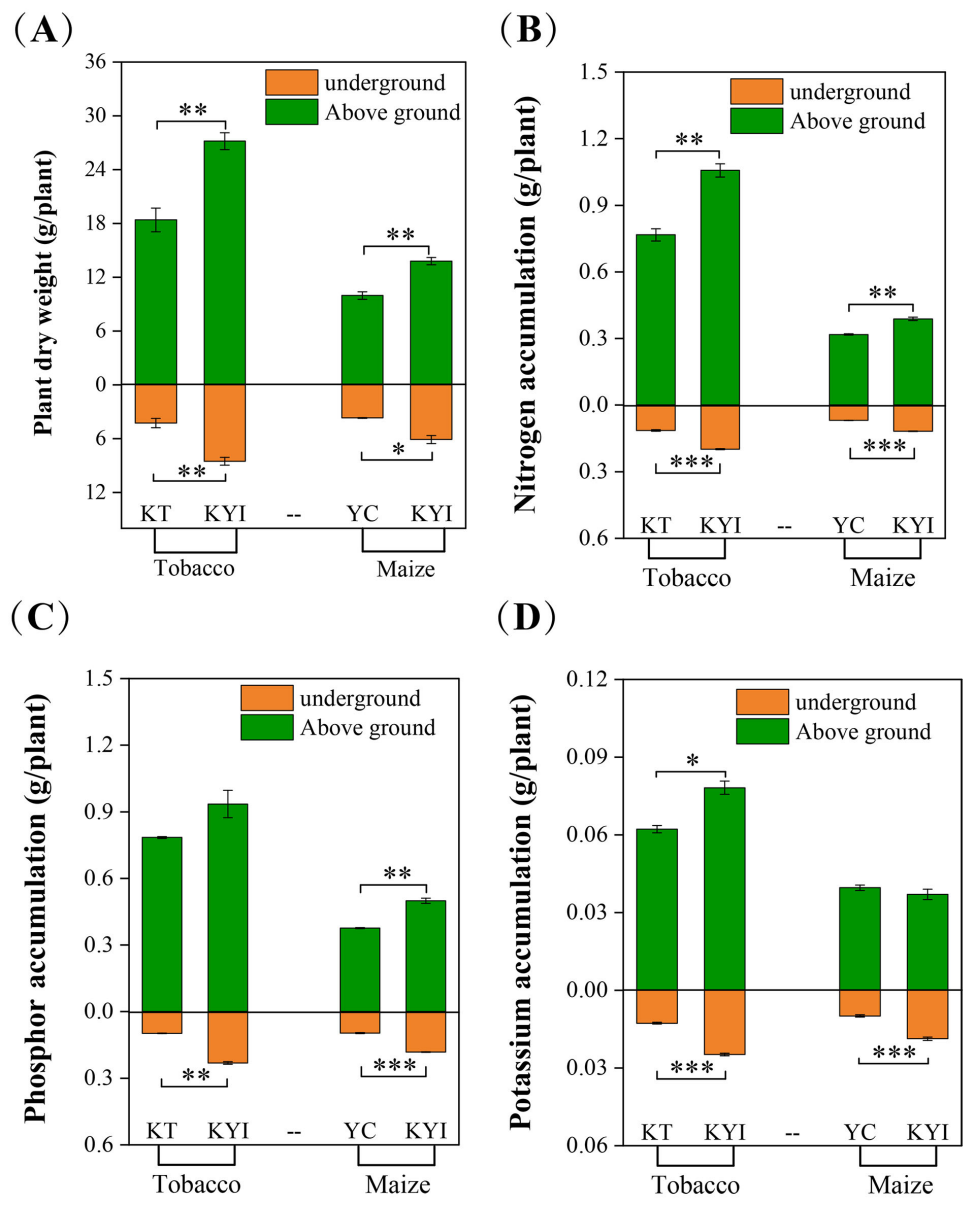
Effects of Intercropping on the Growth of Tobacco and Maize. **(A)** Biomass; **(B)** Nitrogen accumulation; **(C)** Phosphor accumulation; **(D)** Potassium accumulation. *indicates significant differences between the two groups (*p < 0.05, **p < 0.01, ***p < 0.001, independent-samples t-test; n=6). The same as below.

The aboveground and belowground dry weights of tobacco and maize were significantly higher in intercropping than monoculture, the accumulation of nitrogen and phosphorus in the aboveground and belowground parts of tobacco was significantly higher in intercropping than monoculture, a similar trend was observed for potassium accumulation in the underground parts. Likewise, the nitrogen and potassium accumulation in the aboveground and belowground parts of maize were significantly higher in intercropping than monoculture, a markedly higher phosphorus accumulation occurred in the belowground parts (**Figure 1**). These outcomes demonstrate that intercropping is beneficial to increase crop biomass, enhanced the absorption of nitrogen, phosphorus, and potassium in tobacco and maize plants than monoculture.

### 3.2 Effects of intercropping on the rhizospheric soil properties of tobacco and maize

The pH and available potassium content in intercropped tobacco rhizospheric soil were significantly higher than those in monoculture soil. A similar result was observed for the pH and available phosphorus content in maize. The trend of the available phosphorus content in the rhizospheric soil of tobacco and maize was the opposite. The activities of sucrase, catalase, and nitrate reductase in the rhizospheric soil of tobacco, and those of nitrate reductase and acid phosphatase in the rhizospheric soil of maize were significantly higher in intercropping than in monoculture (**Table 1**). This finding indicates that intercropping increased the enzyme activities related to soil nutrients of tobacco and maize.

**Table 1:** Effect of intercropping on agrochemical characteristics and enzyme activity in the rhizosphere soil of tobacco and maize.

Crop		Tobacco		Maize	
Code of treatments		KT	KYI	YC	KYI
pH		4.80 ± 0.04	4.95 ± 0.03*	4.63 ± 0.03	4.95 ± 0.03**
OM	g • kg^-1^	14.08 ± 0.31	13.74 ± 0.24	15.45 ± 0.14**	14.48 ± 0.07
AN	mg • kg^-1^	140.00 ± 6.39	127.75 ± 3.35	136.50 ± 3.50	145.25 ± 3.35
AP	mg • kg^-1^	79.03 ± 6.27**	38.58 ± 0.64	37.76 ± 0.32	131.68 ± 2.52***
AK	mg • kg^-1^	934.60 ± 26.08	1089.70 ± 37.73*	818.70 ± 16.00	845.75 ± 21.95
SC	μmol • d^-1^g^-1^	11.47 ± 0.12	16.11 ± 0.58**	14.37 ± 0.86	13.28 ± 0.82
CAT	μmol • d^-1^g^-1^	112.70 ± 6.73	227.85 ± 16.45**	276.55 ± 6.93**	213.16 ± 7.50
UE	μmol • d^-1^g^-1^	243.79 ± 16.14c	279.08 ± 14.43	200.97 ± 2.91	200.89 ± 9.88
NR	μg • d^-1^g^-1^	1.01 ± 0.03	1.22 ± 0.07*	0.93 ± 0.05	1.45 ± 0.02**
ACP	μmol • d^-1^g^-1^	1591.14 ± 12.56**	1158.82 ± 58.80	1153.42 ± 21.06	1269.57 ± 13.10**

OM, Organic Matter; AN, Available Nitrogen; AP, Available Phosphorus; AK, Available Potassium; SC, Sucrase; CAT, Catalase; UE, Urease; NR, Nitrate reductase; ACP, Acid phosphatase. *represented significant differences of soil properties between monocultures and intercropping patterns for the same crop. (*p < 0.05, ** *p* < 0.01, *** *p* < 0.001).

### 3.3 Effects of intercropping on the rhizosphere soil microbial diversity of tobacco and maize

**Figure 2:**
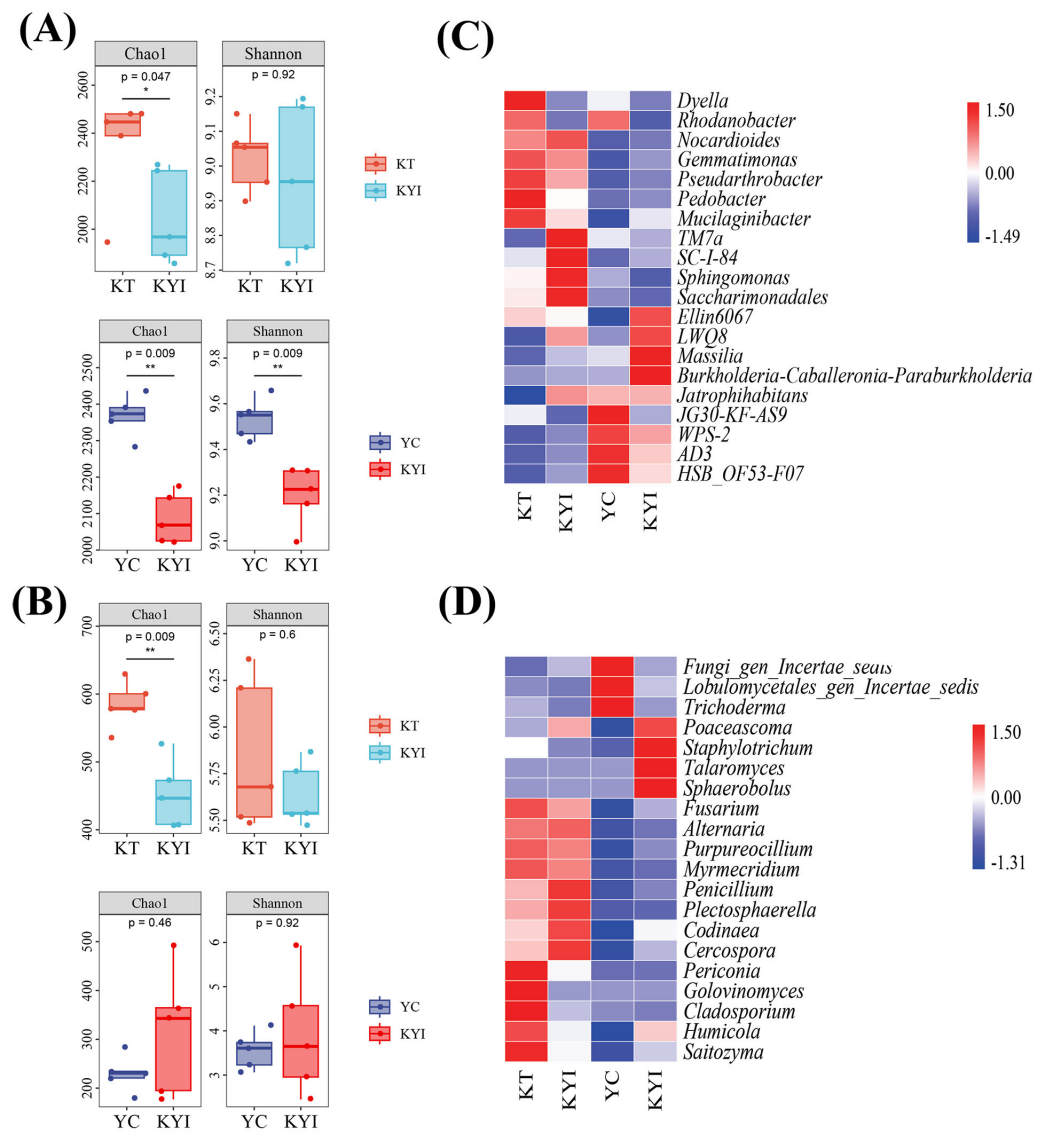
Differences in the structure of microbial communities at different treatments. **(A)** Rhizosphere soil bacterial diversity; **(B)** Rhizosphere soil fungal diversity; **(C)** Heat map of relative abundance of dominant bacteria; **(D)** Heat map of relative abundance of dominant fungi. Alpha diversities of bacteria and fungi in the rhizosphere soil at different treatments, as well as the relative abundances of the top 20 bacterial and fungal species. *indicates significant differences between the two groups (*p < 0.05, **p < 0.01, t-test; n=5). The same as below.

The Chao1 indices of bacteria and fungi in the rhizosphere soil of intercropping tobacco were significantly lower than in the monoculture. The Chao1 and Shannon indices of bacteria in intercropping maize rhizosphere soil were significantly lower than in the monoculture (**Figures 2A, B**). Comparisons were made between the relative abundance of the top 20 bacterial and fungal genera in the tobacco and corn rhizosphere soil. The bacterial genera *Sphingomonas*, *Massilia* and *LWQ8* in the tobacco rhizosphere soil were respectively 3.13%, 1.23%, and 1.19% more abundant in the intercropping treatment than in the monoculture. For maize, the bacterial genera *Gemmatimonas*, *Massilia*, *LWQ8*, and *Burkholderia-Caballeronia-Paraburkholderia* were respectively 1%, 3.60%, 1.24%, and 1.27% more abundant in intercropping than in the monoculture (**Figure 2C**). Moreover, the fungal genus *Penicillium* in the tobacco rhizosphere soil was 7.00% higher in the intercropping treatment than in the monoculture. *Humicola*, *Fusarium*, *Golovinomyces*, and *Cladosporium* were 7.00%, 1.42%, 3.28% and 1.49% lower, respectively, in intercropping than in the monoculture. The fungal genera *Humicola*, *Penicillium* and *Talaromyces* in the maize rhizospheric soil were 9.68%, 3.67%, 30.43% higher, respectively, *Fusarium*, *Trichoderma* were 2.41% and 2.97% lower in intercropping than in the monoculture (**Figure 2D**).

Results indicated that, compared to monoculture, intercropping reduced the richness and diversity of fungi in the tobacco rhizosphere and the richness and diversity of bacteria in the maize rhizosphere, the composition and abundance of bacterial and fungal genera in the rhizosphere soil of tobacco and maize exhibited significant differences between the treatments at the genus level, with generally higher abundance in the intercropping treatment.

### 3.4 Co-occurrence networks of bacterial and fungal communities in the rhizosphere soil of tobacco and maize

**Figure 3:**
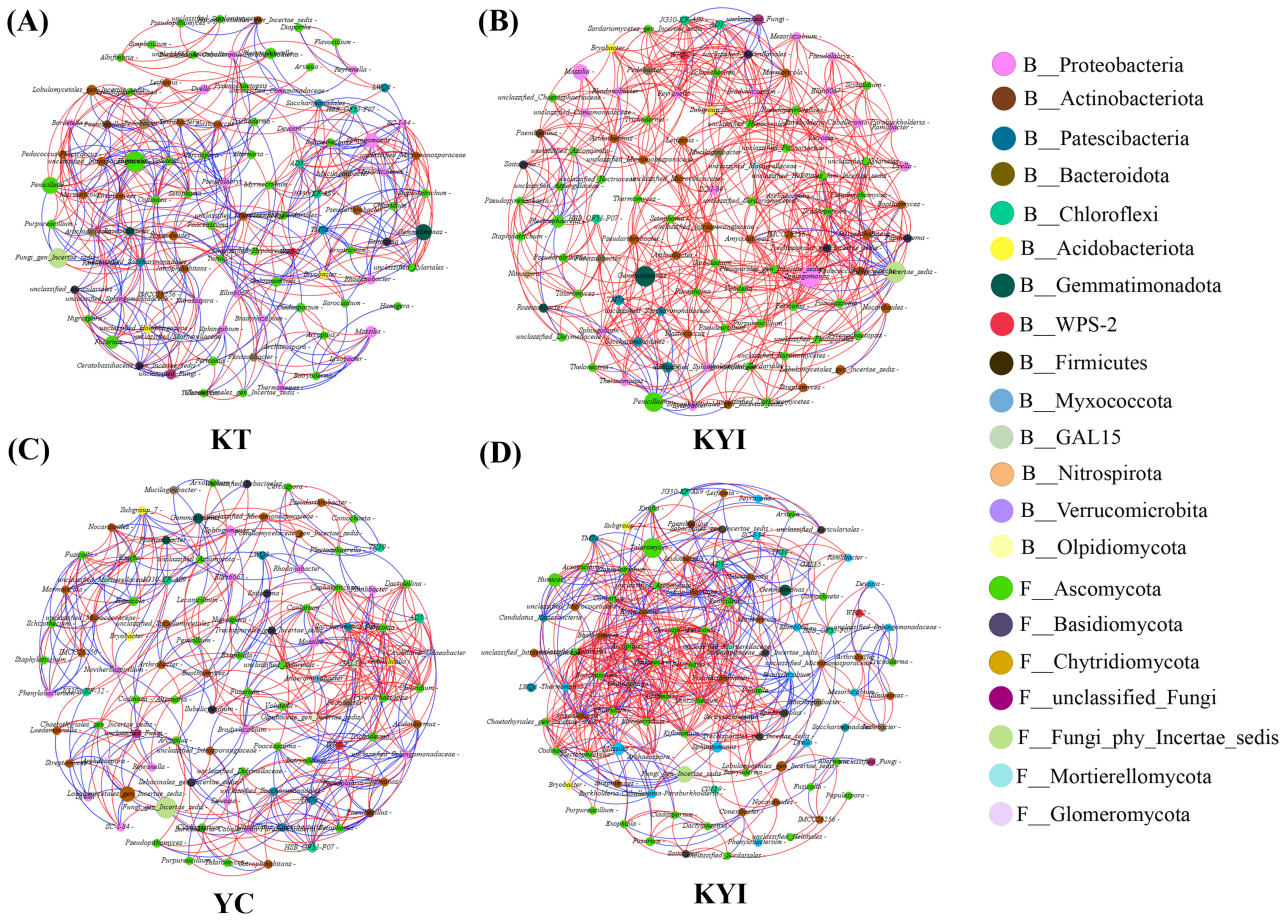
Co-occurrence network of bacterial and fungal communities in the rhizosphere soil of tobacco and maize. **(A)** Single cropping of flue-cured tobacco; **(B)** Intercropping of flue-cured tobacco; **(C)** Single cropping of maize; **(D)** Intercropping of maize. Each node in the network represents a bacterial or fungal genus, and the connection indicates a strong positive correlation with statistical significance (p < 0.05) (red, Spearman’s r > 0.7) or negative correlation (blue, Spearman’s r < - 0.7). Nodes of the same color belong to the same gate, and the thickness of each connection between two nodes is related to the Spearman correlation coefficient, r > 0.7 or < -0.7.

The results of the co-occurrence network analysis of the bacterial and fungal communities of the top 50 genera in the rhizosphere soil of tobacco and maize are shown in **Figure 3** and **Table 2**. The results show different microbial co-occurrence networks for the intercropping and monoculture treatments. The major nodes of the bacterial and fungal communities in the rhizospheric soil include 14 bacterial phyla and 7 fungal phyla. Proteobacteria, Actinobacteriota, and Ascomycota, Basidiomycota, Chytridiomycota were the dominant bacterial and fungal phyla. After intercropping, the co-occurrence network of tobacco and corn increased by 119 and 152 edges respectively compared to monoculture, making the co-occurrence network more complex than monoculture. The relationships for tobacco and maize showed positive correlations of 53.75% and 59.37% in monoculture, respectively, and 92.15% and 68.03% in intercropping. These findings indicate that the symbiotic relationships (positive correlations) between bacterial and fungal communities in the rhizosphere soil of tobacco and maize were stronger than the competitive relationships (negative correlations), suggesting that intercropping improved the relationships among bacterial and fungal genera in the rhizospheric soil.

**Table 2:** Characteristics of bacterial and fungal networks in the rhizosphere soil of tobacco and maize.

Crop	Code of treatments	Modularity	Average clustering coefficient	Average path length	Network diameter	Graph density	Average degree	Nodes	Edges
Tobacco	KT	0.559	0.523	3.653	9	0.097	9.6	100	480
	KYI	0.551	0.598	2.956	7	0.121	11.98	100	599
Maize	YC	0.625	0.549	3.516	7	0.089	8.86	100	443
	KYI	0.451	0.651	3.576	9	0.118	11.7	100	585

### 3.5 Impact of intercropping on the rhizosphere soil metabolites of tobacco and maize

**Figure 4:**
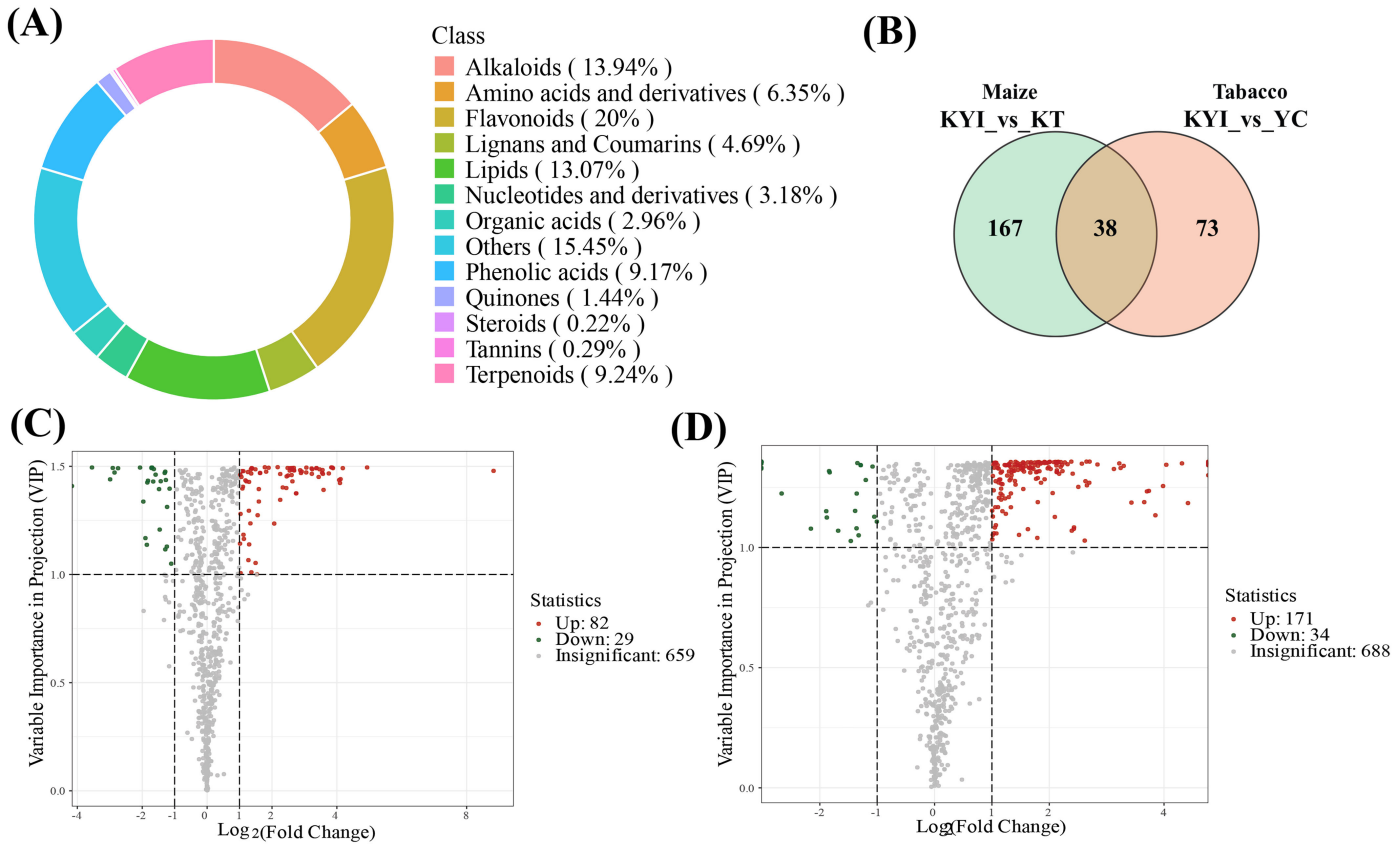
Changes of different metabolites in rhizosphere soil. **(A)** Classification of identified metabolites in rhizosphere soils; **(B)** Venn maps of differential metabolites in rhizosphere soils of tobacco and maize under mono-cropping and intercropping systems; **(C)** Volcanic maps of differentiated metabolites in maize rhizosphere soil (YC vs KYI); **(D)** Volcanic maps of differentiated metabolites in maize rhizosphere soil (KT vs KYI). The panels **(C, D)** are drawn based on the average of three repetitions.

The identified metabolites can be classified into 13 categories, among which flavonoids (20%), others (15.45%), alkaloids (13.94%), lipids (13.07%), and terpenoids (9.24%) are the main metabolites (**Figure 4A**). We further analyzed the differences of special metabolites in rhizosphere soil of four different treatments. Among 688 detected metabolites, 205 were differentially expressed metabolites (DEMs) in the tobacco rhizosphere soil in both treatments. There were 171 upregulated and 34 downregulated DEMs in the rhizosphere soil of intercropped tobacco than monocultural. Similarly, 111 DEMs were identified in maize rhizosphere soil in both treatments among 659 detected metabolites, with 82 upregulated and 29 downregulated DEMs in the rhizosphere soil of intercropped maize (**Figures 4C, D**). To detect the changes of metabolites in the rhizosphere soils of tobacco and maize under monocultural and intercropping systems, we performed a comparative analysis of the differential metabolites in the rhizosphere soils of the two crops and obtained 38 identical differential metabolites (**Figure 4B**; **Supplementary Table S1**). These findings suggest that intercropping significantly influences the metabolite composition in rhizosphere soil and enhances the exudation of root metabolites.

### 3.6 Effects of intercropping on metabolism pathways in rhizosphere soil of tobacco and maize

**Figure 5:**
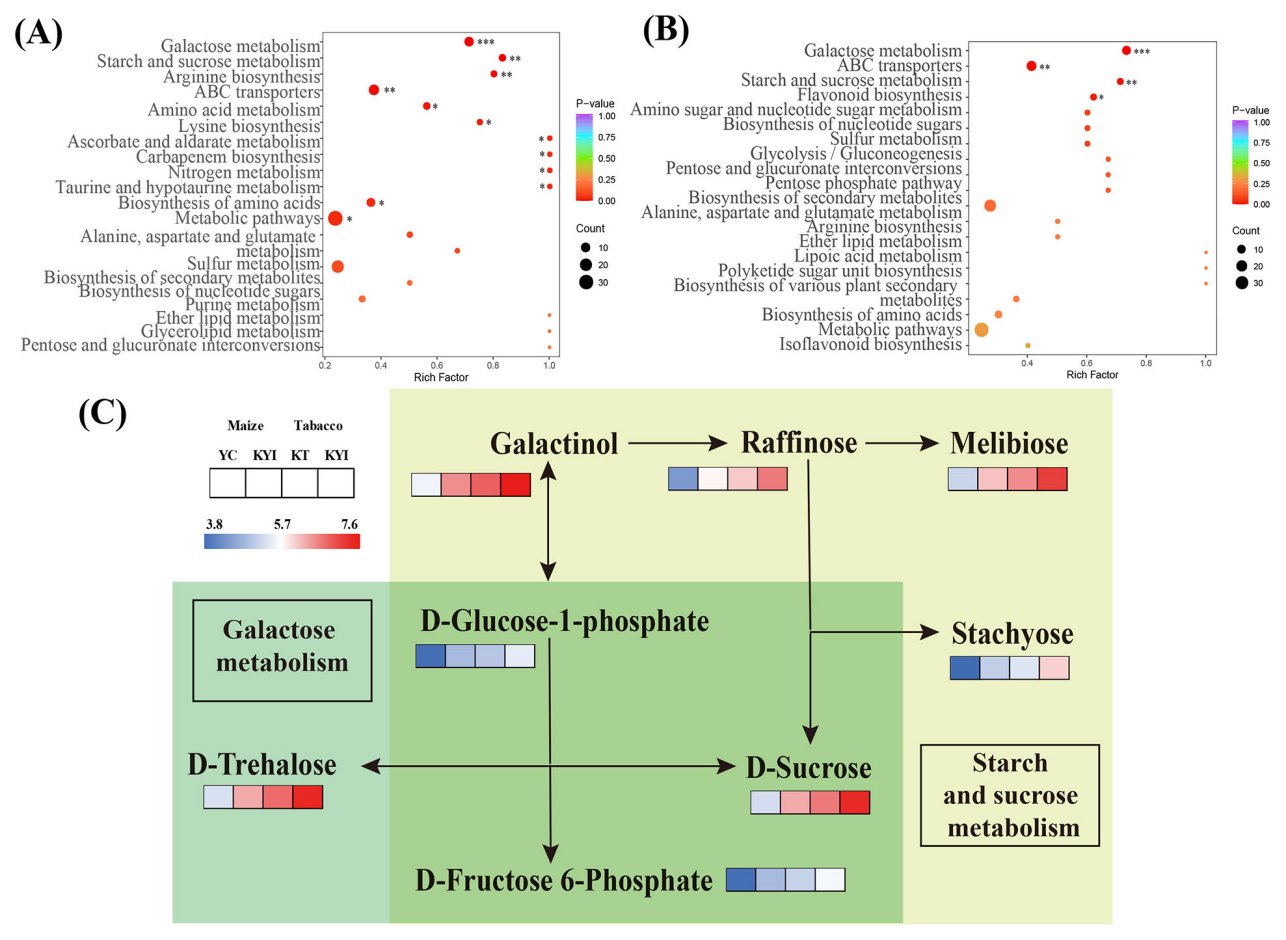
Comparative analysis of metabolites from KEGG enrichment pathway (TOP20) and major pathway of differential metabolites. **(A)** Differential metabolite enrichment pathway between YC and KYI; **(B)** Differential metabolite enrichment pathway between KT and KYI; **(C)** Comparison of major KEGG pathway differential metabolites abundance between intertreatment and single treatment, with blue indicating low content and red indicating high content.

The significantly different metabolites were annotated using the KEGG database (https://www.kegg.jp/). We identified the top 20 pathways with the highest levels of enrichment in both control groups. In the comparison between YC and KYI, Galactose metabolism, Starch and sucrose metabolism, Arginine biosynthesis and ABC transporter were significantly enriched (**Figure 5A**). In the comparison between KT and KYI, Galactose metabolism, ABC transporter, Starch and sucrose metabolism, and Flavonoid biosynthesis were significantly enriched (**Figure 5B**). In the intercropping mode, the changes of carbohydrate metabolism pathways were consistent between the two crops, the metabolite content in all pathways was significantly increased and 8 different metabolites enriched in galactose metabolism pathway and starch and sucrose metabolism pathway were compared and analyzed (**Figure 5C**), and it was found that they were significantly increased in the rhizosphere soil of the two crops in the intercropping mode. D-Glucose-1-phosphate, D-Sucrose, and D-Fructose-6-Phosphate are the core metabolites in both pathways. The results showed that intercropping could increase the content of energy metabolites in rhizosphere soil.

### 3.7 Conjoint analysis

**Figure 6:**
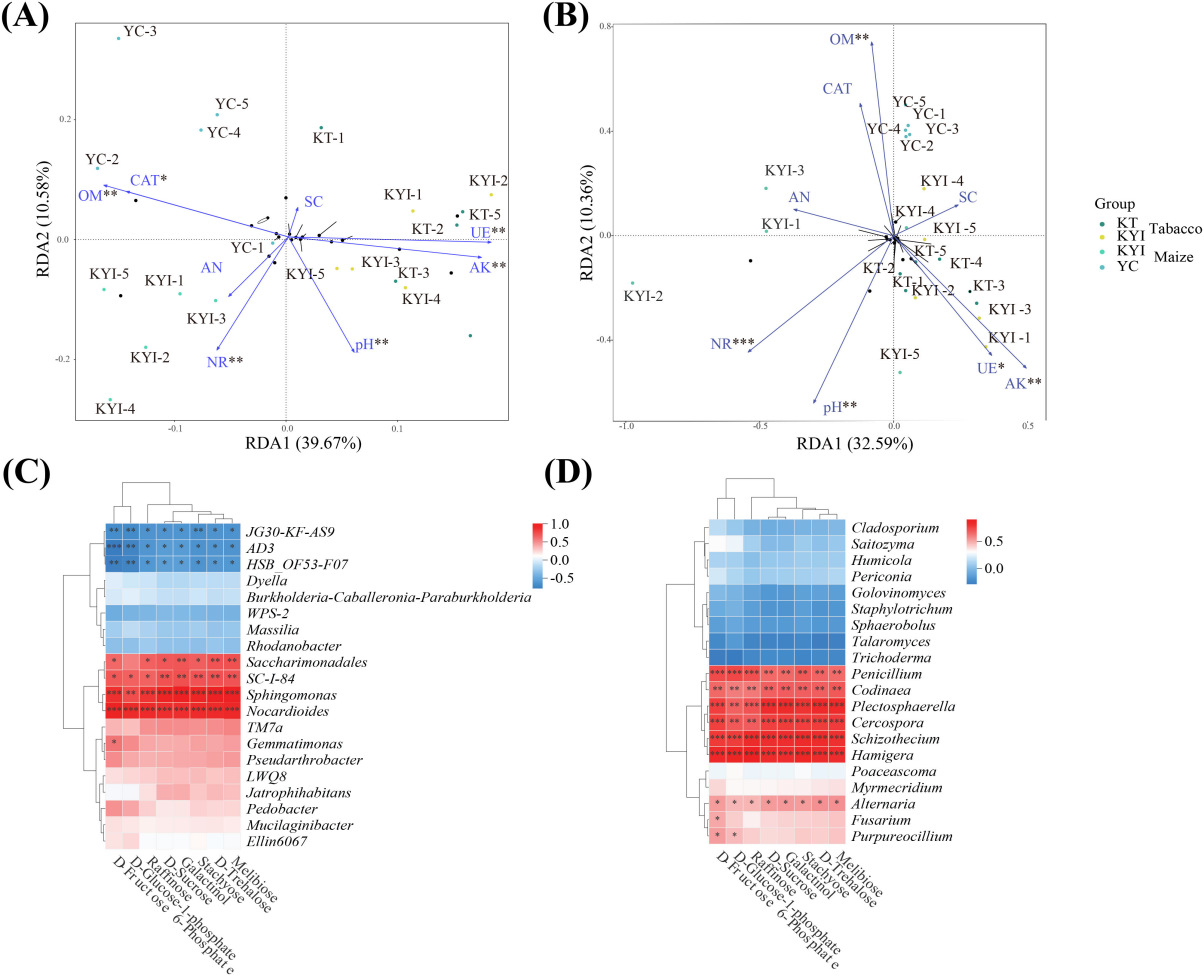
Combine analysis. **(A)** Redundancy analysis of soil bacteria and soil environmental indicators; **(B)** Redundancy analysis of soil fungi and soil environmental indicators; **(C)** Correlation analysis between key metabolites and the abundance of dominant bacteria; **(D)** Correlation analysis of key metabolites with the abundance of dominant fungi. * means *p ≤* 0.05, ** means *p ≤* 0.01, *** means *p ≤* 0.001.

RDA analysis was performed on rhizosphere soil and environmental factors, and two redundant explanatory variables of AP and ACP (VIF > 10) were removed. RDA1 and RDA2 explained 39.67% and 10.58% of the of bacteria community structure, respectively, and 32.59% and 10.36% of the variation were explained by RDA1 and RDA2 on the influence of fungal community structure, respectively (**Figures 6A, B**). In order to study the relationship between differential metabolites in rhizosphere soil and microbial communities, correlation analysis of correlation heat maps was constructed based on the peak area of the top 20 dominant bacteria genera in relative abundance in microbial communities and differential metabolites in key metabolism pathways (**Figures 6C, D**). Bacterial genera *JG30-KF-AS9*, *AD3*, *HSB-OF53-F07* were significantly negatively correlated with differential metabolites, while *Saccharimonadales*, *SC-I-84*, *Sphingomonas*, *Nocardioides* were significantly positively correlated with differential metabolites. The fungi *Penicillium*, *Codinaea*, *Plectosphaerella*, *Cercospora*, *Schizothecium*, *Hamigera* and *Alternaria* were significantly positively correlated with differential metabolites. The results showed that pH, OM, AK, NR and UE were the main environmental factors that significantly affected soil dominant microbial communities. The intercrop pattern exerts a significant impact on the structure of microbial communities by regulating the transformation of carbohydrates in rhizosphere metabolites.

## 4 Discussion

One of the greatest advantage of intercropping is utilizing the interactions between different plants to improve soil physical and chemical properties and enzymatic activities, which promote the growth and development of crops (Gong et al., 2019; Liu et al., 2024; Seerat et al., 2024). In this study, intercropping significantly increased the biomass during the early growth period of tobacco and maize, and promoted the uptake of nitrogen, phosphorus and potassium nutrients by plants. The promotion effect on plant growth is consistent with the previous research results on the tobacco-vegetable intercropping system (Zhou et al., 2023a). On the one hand, intercropping improves the utilization of space, tobacco and maize plant structure significant differences lead to the ground part of the competition for space and sun, can promote the plant photosynthetic (Chen et al., 2023; Keating and Arberry, 1993; Michael et al., 2024), on the other hand, intercropping promotes root growth, improved the absorption and utilization of soil nutrients (Wang et al., 2018).

The interaction between the root systems of maize and tobacco in intercropping affects the nutrient content, enzyme activity (Nasar et al., 2024), microbial diversity (Yang et al., 2024), the types and functionalities of metabolites in the rhizospheric soil, increasing the conversion of organic matter to inorganic matter and facilitating the release of nutrients (Li et al., 2020; Zou et al., 2023). In this study, the pH, available potassium and nitrate reductase activities were significantly higher than those of the control, indicating that intercropping was helpful to improve soil nutrient levels. Alterations in soil nutrients and enzyme activity in intercropping are influenced by soil microbiome (Blagodatskaya and Kuzyakov, 2008; Wang et al., 2021; Zhang et al., 2022).

The richness, diversity of rhizospheric fungi in tobacco and rhizospheric bacteria in maize were lower in intercropping than in monoculture. The possible reason is that tobacco root exudates change the recruitment of rhizosphere microbiome and have a regulatory and inhibitory effect on pathogenic bacteria (Zhou et al., 2023b, Zhang et al., 2019). Therefore, the number of detrimental microorganisms in the rhizospheric soil was lower in intercropping than in the monoculture. Bacterial genera, such as *Gemmatimonas* and *Massilia*, and the fungal genus *Penicillium* were more abundant in the rhizospheric soil of tobacco and maize in intercropping than in the monoculture. *Gemmatimonas*, a multifunctional soil bacterium, promote the absorption and utilization of nutrients and growth of crops, through phosphorus solubilization and nitrogen fixation (Ding et al., 2016). *Massilia* synthesizes various secondary metabolites and enzymes, facilitating phosphorus solubilization and degradation and improving heavy metal tolerance (Yang et al., 2019). Most studies indicated that *Penicillium* showed promise in remediating arsenic-contaminated soil (Lalith et al., 2018; Duan et al., 2019), enhancing the availability of soil nutrients, and promoting plant growth (Yan et al., 2023). The intercropping of tobacco and maize increased the relative abundance of functional microorganisms and decreased the relative abundance of pathogens, such as *Fusarium* in the tobacco rhizosphere, which was beneficial to soil health and crop growth. However, in this study, beneficial microorganisms such as *Humicola* and *Trichoderma* showed lower abundance in intercropping, while the relative abundance of *fusarium* in maize was elevated, indicating an interaction between the roots of maize and tobacco or competition between harmful and beneficial microorganisms (Li et al., 2022).

Differences in the metabolite content were observed between the intercropping and monoculture treatments. The contents of sugars and amino acids in the plant-rhizosphere system were mainly changed by the intercropping system. L-Lysine, Adenine, D-Sucrose, D-Trehalose, and D-Fructose 6-Phosphate were significantly increased in the intercropping mode. This observation is consistent with previous studies on soil intercropping by different plants (Tang et al., 2022). Rhizosphere metabolites are composed of a variety of complex compounds (Hassan et al., 2017), and these soil metabolites are mainly derived from plant root exudates and microbial metabolites (White et al., 2017). Consistent with previous studies, the primary source of metabolites we detected as root exudates (Nishbah et al., 2024), in addition, we also found that the flue-cured tobacco and maize intercropping system regulates the galactose, starch and sucrose metabolism pathway increased the contents of D-Sucrose, D-Trehalose, D-Fructose-6-Phosphate, D-Glucose-1-phosphate in soil, providing an important carbon source and energy source for microorganisms in soil (Xie et al., 2014).

A study demonstrated an intense relationship in the diversity and community composition of microorganisms and the composition of metabolisms in the rhizosphere soil (Shi et al., 2023), root exudates contain chemical signals that mediate plant interactions, modify soil microbial community structure and function, conducive to interspecific interactions, inhibit pathogens, and contribute to soil improvement and ecosystem functions (Yin et al., 2022; Kulmatiski et al., 2008). Rhizosphere metabolites can act as intraspecific and interspecific regulators of microbial communities (Cotton et al., 2019). Thus, these findings highlight the regulation of rhizosphere metabolites on soil microbial communities (Bi et al., 2022; Zhang et al., 2022). The study of Bi et al. (2022) also have shown that some rhizosphere metabolites, such as fatty acids, monoacylglycerides, and diterpenoids can control soil microbial diversity and function. For example, azelaic acid and dicarboxylic acid plays a role in plant-bacterial signaling (Javvadi et al., 2018). As a unique source of nitrogen and phosphorus, azelaic acid can be utilized by bacteria, such as *Pseudomonas* nitride (Janota and Wright, 1964).

In the intercropping system, D-Sucrose, D-Fructose-6-Phosphate, D-Glucose-1-Phosphate1 are metabolites shared with the galactose and starch and sucrose metabolism pathways. There was significant positive correlation with bacteria (*Saccharimonadales*, *Sphingomonas*) and fungi (*Penicillium*, *Codinaea*), and it was enriched in the rhizosphere soil of flue-curing tobacco and maize. These results further indicated that rhizosphere specific metabolites were secreted to recruit rhizosphere specific flora after flue-curing tobacco intercropping with maize, thus changing the original rhizosphere microbial community (Bi et al., 2021; Wang et al., 2023). The marked disparities in the abundance of rhizosphere soil microbiome and the metabolite content suggest that alterations in soil conditions post-intercropping facilitate crop growth and development. Further investigations are needed to identify the key microbial genera and soil metabolites and analyze their synergy and effect on crop growth and soil health. In summary, the study provides a foundation for practical applications in crop cultivation optimization and sustainable agriculture. Through crop intercropping systems, it could enhance crop productivity, promote soil health, and contribute to ecological sustainability. Future research could further explore and optimize specific combinations of crops to maximize these benefits.

## 5 Conclusion

The results demonstrated that intercropping tobacco and maize significantly effects on soil nutrients and enzyme activities compared with monoculture. The intercropping pattern not only increased the biomass and nutrient accumulation of both crops but also promoted beneficial functional microorganisms while suppressing harmful ones. Moreover, intercropping improved soil microbial functions and nutrient availability by altering rhizosphere metabolite composition. These findings underscore the importance of rhizosphere metabolites in harnessing soil microbial communities and nutrient, and regulating crop growth.

In a broader field farming system, the beneficial interactions between tobacco and maize through intercropping can result in higher crop yields, decreased reliance on chemical fertilizers, and improved soil health by improving soil microbes and metabolites. To build on these advantages, future research should prioritize the identification of specific microbial genera and metabolites that play a key role in intercropping systems. This will provide a solid scientific theoretical foundation for developing effective intercropping strategies aimed at enhancing both agricultural productivity and sustainability.

## Data Availability

Raw sequencing data were deposited in the NCBI Sequence Read Archive, https://submit.ncbi.nlm.nih.gov/subs/sra/) with accession No. PRJNA1105019 and No. PRJNA1104889.
