# Supplementary material for: Intercropping of tobacco and maize at seedling stage promotes crop growth through manipulating rhizosphere microenvironment
Source: Front Plant Sci. 2024 Oct 9;15:1470229. doi: 10.3389/fpls.2024.1470229 (PMC11496092; doi:10.3389/fpls.2024.1470229)
Supplement: Supplementary file 1 [file Table1.docx]

Table S1. The contents of different metabolites in rhizosphere of two crops

| Compounds | Class I | YC-1 | YC-2 | YC-3 | KYC-1 | KYC-2 | KYC-3 | KYT-1 | KYT-2 | KYT-3 | KT-1 | KT-2 | KT-3 |
| --- | --- | --- | --- | --- | --- | --- | --- | --- | --- | --- | --- | --- | --- |
| 2-amino-4-methyl-4-pentenoic acid | Amino acids and derivatives | 93931 | 132615 | 93589 | 219142 | 265466 | 195734 | 2398627 | 2262841 | 2394850 | 665825 | 704059 | 840422 |
| DL-Threonine | Amino acids and derivatives | 207102 | 121561 | 124804 | 83608 | 59830 | 49395 | 0 | 0 | 0 | 71179 | 80586 | 77264 |
| L-Lysine | Amino acids and derivatives | 147053 | 111170 | 92833 | 671347 | 715486 | 665051 | 6926794 | 6911319 | 6507564 | 2096205 | 2382871 | 2447778 |
| L-Proline | Amino acids and derivatives | 828522 | 785483 | 666523 | 4664264 | 4602108 | 4189598 | 21870670 | 20672989 | 19324163 | 5722404 | 5782090 | 6000490 |
| L-Citrulline | Amino acids and derivatives | 13229 | 23305 | 1318 | 42896 | 37158 | 29598 | 103249 | 66173 | 113693 | 37117 | 57815 | 46227 |
| L-Glutamine | Amino acids and derivatives | 114747 | 142493 | 120265 | 814624 | 785235 | 806268 | 6918106 | 6941041 | 7046893 | 2221448 | 2387260 | 2557837 |
| Adenine | Nucleotides and derivatives | 748632 | 677200 | 652031 | 1443344 | 1526365 | 1306765 | 1681576 | 1695627 | 1782702 | 838437 | 796322 | 901114 |
| Isopentenyladenine-7-N-glucoside | Nucleotides and derivatives | 2271919 | 2242601 | 2324783 | 5542411 | 6375368 | 5994599 | 6929408 | 7010681 | 6583065 | 2520006 | 2897149 | 2903810 |
| 6-Hydroxy-7-methoxycoumarin | Lignans and Coumarins | 40311 | 60688 | 34202 | 554350 | 521715 | 515271 | 4116819 | 3906958 | 4250309 | 10033047 | 9113815 | 10832360 |
| D-Maltose | Others | 291702 | 301921 | 291007 | 4101287 | 4660036 | 3805378 | 28892248 | 28695959 | 29760896 | 6263474 | 6635801 | 6080725 |
| D-Melezitose | Others | 21616 | 15068 | 20356 | 155209 | 143803 | 117397 | 3087947 | 2772226 | 3827007 | 529894 | 489422 | 614391 |
| D-Panose | Others | 5315 | 2517 | 4143 | 40064 | 54710 | 45160 | 905645 | 694095 | 1295013 | 107520 | 95303 | 91719 |
| D-Pinitol | Others | 524978 | 567019 | 603604 | 44562 | 53360 | 47090 | 0 | 0 | 0 | 21317 | 45170 | 23905 |
| D-Sucrose | Others | 98603 | 127857 | 102609 | 1156509 | 1205826 | 1047401 | 19227538 | 18668891 | 18892152 | 3072007 | 2721830 | 2933154 |
| D-Trehalose | Others | 108221 | 146743 | 109982 | 1211202 | 1095724 | 1094781 | 20138419 | 19747579 | 20181771 | 3970889 | 3511002 | 4027574 |
| Galactinol | Others | 175079 | 223378 | 166998 | 2040009 | 2020449 | 1755649 | 31675978 | 30569175 | 29694911 | 4966760 | 4504912 | 5195195 |
| Isomaltulose | Others | 264411 | 275533 | 220391 | 4512316 | 5417796 | 3868435 | 28393801 | 32113617 | 32886703 | 7047553 | 6762100 | 7435075 |
| Isovanillin | Others | 113782 | 138341 | 105585 | 233138 | 272239 | 239333 | 712196 | 726806 | 715589 | 153374 | 156010 | 137150 |
| Maltotriose | Others | 1269512 | 1297519 | 1212795 | 4365132 | 4371216 | 4231588 | 20491527 | 12692833 | 27155383 | 5058056 | 5326366 | 5583171 |
| Melibiose | Others | 82553 | 99595 | 88746 | 790673 | 752744 | 714259 | 9000691 | 10439760 | 10593651 | 1674946 | 2002846 | 2342783 |
| Nystose | Others | 22399 | 24819 | 25663 | 88526 | 90985 | 91612 | 1409668 | 1012469 | 1268389 | 239836 | 246591 | 304651 |
| Raffinose | Others | 22900 | 19083 | 20993 | 318884 | 314842 | 276628 | 2497250 | 2168733 | 4002264 | 672196 | 583558 | 726404 |
| Stachyose | Others | 6282 | 9421 | 3826 | 68430 | 78492 | 84758 | 562648 | 515744 | 645081 | 128660 | 136742 | 129918 |
| Vanillin; 4-Hydroxy-3-Methoxybenzaldehyde | Others | 148052 | 146328 | 129613 | 273843 | 312117 | 309793 | 837982 | 856391 | 849718 | 198399 | 174249 | 197720 |
| (S)-2,3-dihydroxypropyl (9Z,12Z,15Z)-octadeca-9,12,15-trienoate | Others | 73391 | 64238 | 69784 | 162242 | 211359 | 134128 | 346963 | 763625 | 707892 | 303656 | 286451 | 287805 |
| 5-(2-hydroxypropyl)-3H-2-benzofuran-1-one | Others | 166531 | 129495 | 41995 | 1855298 | 2122015 | 1870682 | 20016805 | 21966193 | 22351355 | 5852962 | 6156406 | 8572988 |
| D-Cellobiose | Others | 6926 | 4393 | 3145 | 50302 | 45853 | 39296 | 568716 | 610468 | 488371 | 146218 | 91503 | 125618 |
| D-Fructose 6-Phosphate | Others | 4894 | 13880 | 2978 | 53118 | 47536 | 45158 | 182938 | 157192 | 268253 | 63708 | 105519 | 90375 |
| D-Glucose-1-phosphate | Others | 4894 | 13880 | 2978 | 53118 | 47536 | 45158 | 146603 | 94917 | 216713 | 56555 | 78660 | 58518 |
| Laminaran | Others | 6281 | 4399 | 1275 | 64666 | 83126 | 56502 | 765909 | 520889 | 1110442 | 155931 | 123120 | 172681 |
| Maltitol | Others | 12953 | 11768 | 6824 | 91674 | 87871 | 82496 | 850579 | 844392 | 1031311 | 242099 | 236649 | 277546 |
| 4-Methylazetidine-2-Carboxylic acid | Alkaloids | 492131 | 509381 | 413241 | 3046661 | 2858408 | 2775965 | 16825414 | 16117687 | 15596854 | 3598184 | 3764849 | 3892461 |
| Imidazole-4-Acetic Acid | Alkaloids | 165812 | 159746 | 174613 | 325596 | 394105 | 365580 | 711509 | 636548 | 721810 | 221808 | 268557 | 292446 |
| Grossamide | Alkaloids | 19104 | 26603 | 22276 | 372981 | 309458 | 317556 | 4477667 | 4747379 | 4440136 | 1312619 | 1039889 | 1154583 |
| 7,13,15-Abietatrienoic acid | Terpenoids | 5541 | 5521 | 7218 | 21152 | 18561 | 16892 | 7090 | 8358 | 13261 | 0 | 0 | 0 |
| 2-Aminoethanesulfonic acid | Organic acids | 8790 | 6910 | 4593 | 39429 | 35626 | 48339 | 410604 | 419580 | 410023 | 159648 | 158923 | 175158 |
| Choline Alfoscerate | Lipids | 38142 | 38350 | 30430 | 128324 | 98005 | 105553 | 1493358 | 1098529 | 2115042 | 347177 | 320243 | 336535 |
| Glycerol 9,11,13-octadecatrienoyl ester | Lipids | 38958 | 41505 | 39877 | 121527 | 100056 | 116148 | 455020 | 95398 | 91074 | 46654 | 36571 | 36066 |
